# Supplementary material for: Role of Rab13, Protein Kinase A, and Zonula Occludens-1 in Hepatitis E Virus Entry and Cell-to-Cell Spread: Comparative Analysis of Quasi-Enveloped and Non-Enveloped Forms
Source: Pathogens. 2024 Dec 20;13(12):1130. doi: 10.3390/pathogens13121130 (PMC11678111; doi:10.3390/pathogens13121130)
Supplement: Supplementary file 1 [file pathogens-13-01130-s001.zip › pathogens-3297541-supplementary.pdf]

Supplementary Table S1. The primers used for semi-quantitative RT-PCR.

Target gene	Size	Forward primer (5' – 3')	Reverse primer (5' – 3')
CLDN1	468 bp	CAGTGGAGGATTTACTCCTATGCC	ACAGCAAAGTAGGGCACCTCC
CLDN2	636 bp	ACACTGGTTGCCATGCTGCTCC	TCTTCACACATACCCTGTCAGGC
CLDN3	997 bp	CACCATCGTGTGCTGCGC	TGCAAAACGAAAGGCTTTTATTGA
CLDN4	591 bp	CTTCCAGGTCCTCAACTCCC	GGACAGTTGCAGCAAAGCAGC
CLDN5	740 bp	CGTCTCGCCTCTAGCCATG	CTCCCCAGGCTTATCCAACG
CLDN6	576 bp	TTGCTGGTCTACCTTGCTGG	GGTGAGAAACAGCAGAGCCT
CLDN7	450 bp	TTCATCGTGGCAGGTCTTGCC	TCTTTTTGTCTCTCCCACCAACGG
CLDN8	994 bp	GCTGTCACTGTCATGCCTCA	ACCATATAAGCATGTCTCTATGTGA
CLDN9	695 bp	CTAACCGAGGGGCCAGATG	TGGGCTCCCAGGGGAAAC
OCLN	527 bp	AGTCCCATGGCATACTCTTCC	TTACTTTTGTAATCTGCAGATCCC
ZO-1	886 bp	AGATAGTTTGGCAGCAAGAGATGGC	ACGATAAACATCCTTCTTCTTCTGAGCC
ZO-2	1,449 bp	CGAAGAGTATGGTCTCCGGC	TCCACAGCTTTCGGAGTCAC
ZO-3	1,950 bp	GGAGCTGACCATCTGGGAAC	GTCCTGGACACAGTCTCTGC
JAM-A	704 bp	AGACACCACCAGACTCGTTTGC	AGGCTCACACCAGGAATGACG
JAM-B	595 bp	TTAGAGTGGAAGAAACTGGGTCGG	GCCACAAACGGAAATCACTAAGGC
JAM-C	539 bp	CTTTATCGCTGTGAGGTCGTTGC	ACTCCATCTGGTTTCCCTGGG
GAPDH	564 bp	TCATCCATGACAACTTTGGTATCGTG	CTCTTCCTCTTGTGCTCTTGCTGG

CLDN, Claudin; OCLN, Occludin; ZO, Zonula occludens; JAM, Junctional adhesion molecule; GAPDH, glyceraldehyde-3-phosphate dehydrogenase.
